# Invasive Eye Infection Caused by *Trichoderma Harzianum*

**DOI:** 10.4269/ajtmh.24-0037

**Published:** 2025-01-07

**Authors:** Fei Han, Jiaogui Ou, Na Huang

**Affiliations:** ^1^ Department of Clinical Laboratory, Chongqing University Three Gorges Hospital, Chongqing, China;; ^2^ Department of Ophthalmology, Chongqing University Three Gorges Hospital, Chongqing, China

## Abstract

*Trichoderma* is a widely distributed thermophilic fungus that grows on moist soil, fallen leaves, and rotten wood. It plays an important role in agricultural production, food processing, and soil. However, some forms of *Trichoderma* can infect humans. Aggressive infections are more common in immunocompromised patients, with manifestations ranging from focal to disseminated infections. Here, we report a case of an invasive eye infection in China. The patient, a healthy 64-year-old man, was inadvertently struck by a puncture vine, injuring his eye and resulting in reduced visual acuity, lacrimation, and redness in the right eye. Upon admission, he was diagnosed with right eye perforation injury, right eye iris damage, right eye vitreous opacity, and right eye infection. After completion of the relevant auxiliary examinations, the diagnosis was confirmed by matrix-assisted laser desorption/ionization time-of-flight mass spectrometry and metagenomic next-generation sequencing, and the patient responded to antifungal therapy.

## INTRODUCTION

Endophthalmitis is a vision-threatening emergency, for which timely treatment can significantly affect the final outcome.^1^^,^^2^ On the one hand, examination of the patient’s history and symptoms includes an assessment of visual changes, ocular pain, and inflammatory findings. On the other hand, risk factors include ocular trauma or surgery, immunocompromised status, diabetes mellitus, and the use of injected drugs.^1^^,^^3^ Bacteria and fungi are mostly responsible for endogenous endophthalmitis, with fungal infections causing up to 50% of all cases, of which *Candida albicans* (yeast) and *Aspergillus* (mold) represent the most common causative agents.^4^

*Trichoderma* species are saprophytic fungi commonly found in humid soils and decaying wood, and they are not usually considered pathogens in healthy individuals.^4^^,^^5^ It is worth noting that* Trichoderma* species have been recently reported as being among emerging fungal pathogens, causing a variety of infections, including endophthalmitis, endocarditis, invasive sinusitis, cutaneous infections, mediastinitis, peritonitis, liver infection, stomatitis, and disseminated infections.^5^^,^^6^ Here, we report a rare case of endophthalmitis caused by *Trichoderma harzianum* in a patient with acute ocular trauma.

## CASE REPORT

While logging, a 64-year-old healthy Asian male farmer was inadvertently struck by the back of a puncture vine, resulting in reduced visual acuity, lacrimation, and redness in his right eye. He had a history of chronic heavy smoking and alcohol consumption spanning four decades, but he did not present with any other significant comorbidities, such as diabetes mellitus, hypertension, malignancy, or AIDS.

**Figure 1.**
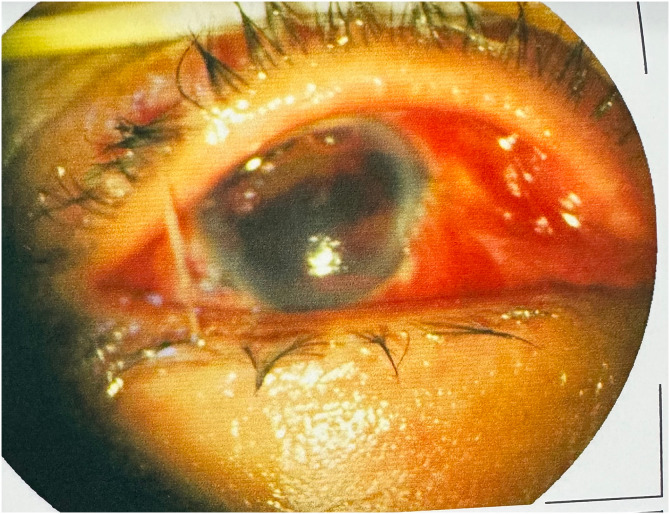
An anterior segment photo of the right eye in admission.

During hospitalization, his right eye was able to count fingers at 30 cm. Anterior segment photography of the right eye showed a shallower anterior chamber, with iris tissue entrapment within the eyeball-penetrating injury, the formation of membranous exudates, and pupil displacement and deformation as well as lens opacities (Figure 1). Laboratory investigations showed that the results of the coagulation test, procalcitonin and C-reactive protein levels, markers of liver and kidney function, and indicators of infectious disease were all in the normal range, whereas the neutrophil-to-lymphocyte ratio was elevated.

The attending doctor diagnosed ocular trauma and performed ocular surgical treatment (the right eye underwent debridement and suturing together with injections of intravitreal vancomycin hydrochloride and ceftazidime), taking a membranous exudate for fungal culture. The patient was placed on an intravenous drip of systemic, empirical, and broad-spectrum antibiotics (ceftazidime and vancomycin). After the operation, an assessment of his right eye showed poor best-corrected visual acuity (BCVA) with light perception. The intraocular pressure (IOP) values were 16 and 19 mmHg in the left and right eyes, respectively. A slit lamp examination of the right eye revealed moderate conjunctival congestion and corneal edema. A brightness scan ultrasound of the right eye showed vitreous hemorrhage.

**Figure 2.**
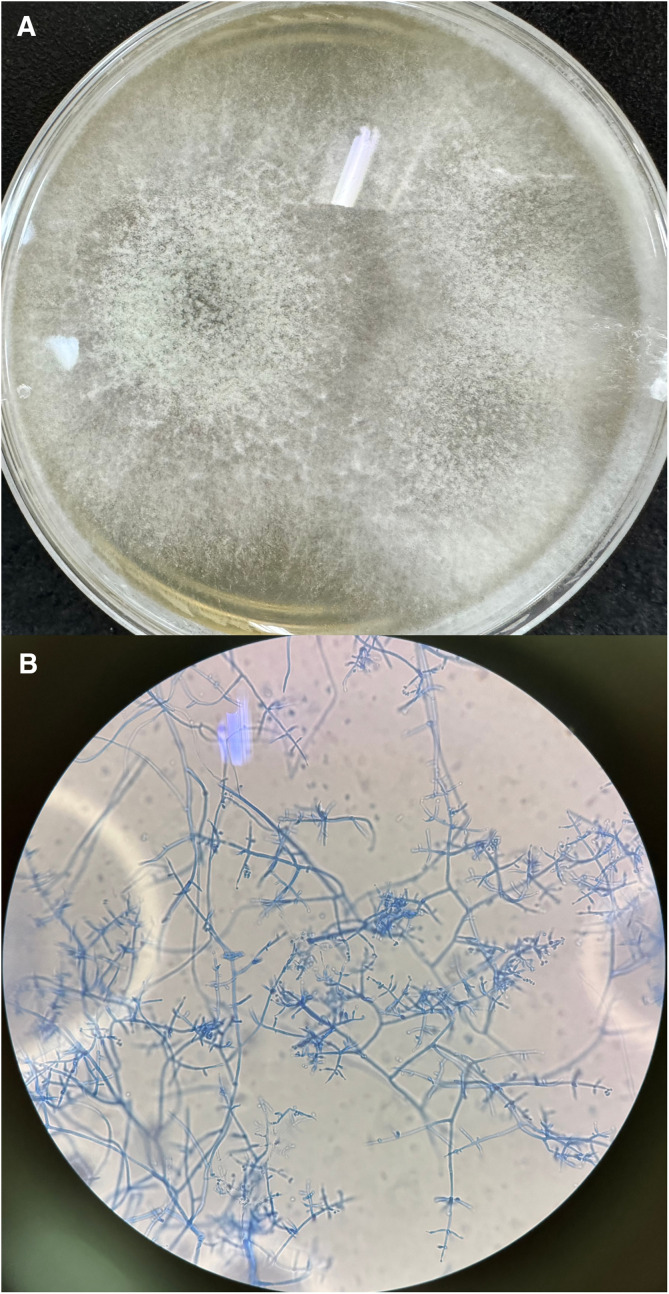
(**A**) Macroscopical features of *Trichoderma harzianum* obtained after 5 days of culture on Sabouraud dextrose at 25°C. (**B**) Microscopical aspects of *T. harzianum*. Magnification: ×1,000.

The culture of the membranous exudate from the right eye was performed at the Microbiology Laboratory of Chongqing University Three Gorges Hospital, with incubation on Sabouraud’s dextrose agar at 25°C. This showed the presence of a filamentous fungus after 5 days of culture (Figure 2A). Staining of the fungal culture with lactophenol cotton blue (Figure 2B) was positive. Matrix-assisted laser desorption/ionization time-of-flight mass spectrometry and metagenomic next-generation sequencing (mNGS) showed a high abundance and sequence number of *T. harzianum* in the fungal culture. Subsequently, intravenous fluconazole was initiated by the doctor as systemic antifungal monotherapy, and it was continued for 5 days. This led to a gradual improvement in the symptoms, and the final ophthalmic evaluation showed mild conjunctival congestion, mild corneal edema, a small amount of bleeding in the vitreous body, and distance sine correctore (DSC) oculus dexter (OD) 4.5/30. The IOP values were 13 and 9 mmHg in the left and right eyes, respectively. The patient was discharged and followed up on an outpatient basis.

## DISCUSSION

Endophthalmitis is a potentially devastating intraocular infection.^1^ Exogenous endophthalmitis is more common than endogenous endophthalmitis, and it is secondary to recent ocular trauma or ophthalmic interventions, such as surgery or injections.^1^^,^^7^ To the best of our knowledge, endophthalmitis caused by *T. harzianum* in a healthy patient has not been reported in the literature. *T. harzianum* is an uncommon opportunistic pathogen belonging to the genus *Trichoderma*, and it is usually found in humid soil and decaying wood. Infections can lead to mortality rates of up to 53% in immunocompromised patients.^6^^,^^8^

For patients with *T. harzianum* infections, doctors need to combine the patient’s history and physical examination to initiate appropriate treatment immediately, whereas cultures of tissue samples are needed for a definitive diagnosis of a *Trichoderma* infection.^9^ Fungal culture and mNGS are effective in the diagnosis of infections caused by rare pathogens.^10^^,^^11^

There are few reported cases of *T. harzianum* infections in healthy humans, and here, we report the case of ocular fungal infection caused by *T. harzianum* in a healthy patient. We are guessing that the infection was because of accidental trauma from a puncture vine that was hypothetically carrying the fungus, and Wang and Tu also reported that the *Trichoderma longibrachiatum* culture of the planting soil was positive (the patient had a history of close contact with a pot of *Epipremnum aureum* for more than 1 month before the onset of the disease).^5^ Bhullar et al. reported that among the exogenous causes, penetrating eye injury (56.3%) was the most common etiological factor.^7^ The effective treatment of fungal endophthalmitis rests on the combination of intravitreal antibiotics and systemic antifungal therapy.^7^^,^^12^

In conclusion, the report indicates that fungal infections caused by the *Trichoderma* species can also occur in individuals with normal immunity. Therefore, it is recommended that doctors pay attention to the presence of fungal infections after injuries by plants during the diagnosis and treatment process.

## References

[1] Cunningham C Widder J Raiji V, 2017. Endophthalmitis. Dis Mon 63: 45–48. 27863689 10.1016/j.disamonth.2016.09.005

[2] Das T Joseph J Simunovic MP Grzybowski A Chen K-J Dave VP Sharma S Staropoli P Flynn H, 2023. Consensus and controversies in the science of endophthalmitis management: Basic research and clinical perspectives. Prog Retin Eye Res 97: 101218. 37838286 10.1016/j.preteyeres.2023.101218

[3] Gunalda J Williams D Koyfman A Long B, 2023. High risk and low prevalence diseases: Endophthalmitis. Am J Emerg Med 71: 144–149. 37393773 10.1016/j.ajem.2023.06.029

[4] Ness T Pelz K Hansen LL, 2007. Endogenous endophthalmitis: Microorganisms, disposition and prognosis. Acta Ophthalmol Scand 85: 852–856. 17725616 10.1111/j.1600-0420.2007.00982.x

[5] Wang S Tu J, 2023. Invasive pulmonary infection caused by *Trichoderma longibrachiatum*. Thorax 78: 632–633. 36754640 10.1136/thorax-2022-219855

[6] Al-Shehri A Aljohani S Semidey VA, 2021. Bilateral endogenous *Trichoderma endophthalmitis* in an immunocompromised host. Am J Ophthalmol Case Rep 24: 101234. 34816055 10.1016/j.ajoc.2021.101234 PMC8592872

[7] Bhullar GK Dawkins RCH Paul RA Allen PJ, 2020. Fungal endophthalmitis: A 20-year experience at a tertiary referral centre. Clin Exp Ophthalmol 48: 964–972. 32639080 10.1111/ceo.13820

[8] Harman GE Howell CR Viterbo A Chet I Lorito M, 2004. *Trichoderma* species—Opportunistic, avirulent plant symbionts. Nat Rev Microbiol 2: 43–56. 15035008 10.1038/nrmicro797

[9] De Miguel D Gómez P González R García-Suárez J Cuadros JA Bañas MH Romanyk J Burgaleta C, 2005. Nonfatal pulmonary *Trichoderma viride* infection in an adult patient with acute myeloid leukemia: Report of one case and review of the literature. Diagn Microbiol Infect Dis 53: 33–37. 15994049 10.1016/j.diagmicrobio.2005.04.009

[10] Austin B, 2017. The value of cultures to modern microbiology. Antonie Van Leeuwenhoek 110: 1247–1256. 28168566 10.1007/s10482-017-0840-8

[11] Han D Li Z Li R Tan P Zhang R Li J, 2019. mNGS in clinical microbiology laboratories: On the road to maturity. Crit Rev Microbiol 45: 668–685. 31691607 10.1080/1040841X.2019.1681933

[12] Modjtahedi BS Finn AP Barb SM MacLachlan MJ van Zyl T Papakostas TD Eliott D, 2019. Characteristics and outcomes of endogenous endophthalmitis: Eight-year experience at a tertiary care center. Ophthalmol Retina 3: 61–72. 30929817 10.1016/j.oret.2018.08.009

